# The clinical analysis and treatment strategy of endovascular treatment for cerebral venous sinus thrombosis combined with intracerebral hemorrhage

**DOI:** 10.1038/s41598-020-78570-1

**Published:** 2020-12-18

**Authors:** Xin-bin Guo, Shuo Liu, Sheng Guan

**Affiliations:** Department of Interventional Radiology, The First Affiliated Hospital of Zhengzhou University, 1 Jianshe Road, Zhengzhou, 450052 China

**Keywords:** Neuroscience, Neurology

## Abstract

Cerebral venous sinus thrombosis (CVST) combined with intracerebral hemorrhage(ICH) is a special subgroup, and whether intrasinus thrombolysis (IST) or mechanical thrombectomy (MT) therapy should be carried out is controversial because of previous hemorrhage and possible delayed hemorrhage.The aim of this study was to analyze the safety and effectiveness of endovascular treatment of CVST with ICH and further discuss the treatment strategy. This is a retrospective study of 56 cases admitted from January 2010 to June 2019 diagnosed with CVST combined with ICH, and all were treated with endovascular treatment including IST with or without MT. We reviewed the clinical, radiological, and outcome data of all patients. The percentage of cases that showed complete and partial recanalization were 67.8% and 26.9% after endovascular treatment. ICH exacerbation occurred in 5 cases during thrombolysis. The percentage of cases with good outcome was 87.5% at discharge. 51 cases were followed up at sixth month. 49 cases had a mRS score of 0–2,and 2 cases had a mRS score of 3–4 at sixth month.Our data suggest that endovascular treatment may improve clinical and radiological outcome in most patients of CVST with ICH, but confirmation in prospective, controlled studies is warranted.

## Introduction

Cerebral venous sinus thrombosis (CVST) is a rare variant of stroke. The symptoms and clinical outcome are highly variable. At least 13% of patients with CVST experienced poor outcomes even after treatment with anticoagulation^1–3^. Several risk factors for such poor outcome have been identified, including coma, intracerebral hemorrhage (ICH), and thrombosis of the deep venous system^4,5^. One of the most significant complications of CVST is ICH, and ICH is a poor prognostic factor and a major cause of mortality among patients with CVST^4,5^.

The initial treatment for CVST is systemic anticoagulation^6^. ICH is one of the devastating potential complications of anticoagulation therapy, especially for the patients with prior history of ICH. CVST combined with ICH is usually classified as severe venous sinus thrombosis^7,8^. ICH is usually associated with poorer outcomes compared to CVST alone without ICH. Without effective treatment, it may leads to deteriorated clinical course, often with poor prognosis or even death^7–10^. Considering the possibility of further aggravation of the ICH, hemorrhagic CVST becomes a therapeutic challenge. Endovascular intervention may be an alternative therapy for hemorrhagic CVST patients. Although great progress has been made in the treatment of CVST, but comphrehensive study of CVST with ICH are limited and current literature only provide case study describing specific technique^11,12^. In our retrospective study of a series of hemorrhagic CVST patients, we analyzed the safety and effectiveness of endovascular interventional therapy for CVST with ICH and further discuss the treatment strategy of CVST with ICH.

## Methods

### Patients selection

Patients included in this retrospective cohort study were selected in the Department of Interventional Neuroradiology of our hospital from January 2010 to June 2019. Datas were recorded including demographic data, clinical symptoms and signs, radiological outcome (MRI, MRV, DSA), treatments in hospital, and complications. The study was approved by the Ethics Committee of the First Affiliated Hospital of Zhengzhou University. All methods were performed in accordance with the relevant guidelines. All patients or their legally authorized representatives signed an informed consent form.

Diagnosis of CVST was based on the clinical and neurologic findings and confirmed by imaging techniques, including brain computed tomography (CT) and/or magnetic resonance imaging (MRI), and magnetic resonance venography (MRV) with or without digital sub-traction angiography (DSA). Anticoagulation with conventional heparin was the initial treatment in all patients. The anticoagulation treatment consisted of heparin 1 Ug/kg subcutaneously injected once every 12 h^5^ .Partial thromboplastin time (PTT) monitoring was performed to adjust the dose of heparin. Inclusion criterias were as follows: (1)CVST was confirmed by the relevant clinical and imaging datas; (2) Diagnosis of ICH by CT or MRI; (3)Symptoms have not been improved or neurological deficits are rapidly deteriorated after anticoagulation treatment; (4) Endovascular interventional therapy was performed ; (5)The clinical datas were complete and there were follow-up datas; (6) patients’ consents were obtained.

### Procedure

The procedure of local thrombolytic therapy is as follows. The location of venous sinus thrombosis was confirmed by DSA after systemic heparinization. The microcatheter was introduced into the distal end of the thrombosed sinus. Continuous urokinase (42,000 U/h, total 1,000,000 U per day) was administered into the cerebral venous sinus by microcatheter. On the 7th day of thrombolysis, DSA or MRV was performed to evaluate the CVST recanalization. If the CVST was not recanalized, the position of the microcatheter needs to be adjusted and urokinase needs to be administered continuously. Infusion was continued until significant clinical improvement or partial recanalization of the sinus with good outflow is seen. All patients in the thrombolysis group were subsequently started on oral anticoagulation for 6 months (warfarin therapy), and International Normalized Ratio (INR) was maintained between 2.0–3.0.

The procedure of mechanical thrombectomy using Solitaire stent retriever combined with local thrombolysis was as follows. A 8 Fr guiding catheter was navigated into the right internal jugular vein. A 2.7 Fr excelsior microcatheter was placed in the anterior portion of the SSS. Then, the Solitaire FR (6 × 20 mm) was deployed in the anterior portion of the SSS, and it was left for five minutes. We attempted the same procedures until DSA revealed partial recanalization of the venous sinus . Then, we placed a microcatheter into venous sinus for local thrombolysis. The procedure of local thrombolytic therapy was described above. Balloon thrombectomy and aspiration thrombectomy was also used in several cases.

The use of the systemic anticoagulant was according to the guidelines of American Heart Association/American Stroke Association. Warfarin was maintained for 6 months at least. International normalized ratio (INR) was dynamically monitored to adjust the dose of warfarin for a target INR of 2–3.

### Clinical events

Any clinical events occurred in the thrombolysis course were noted. Clinical neurologic assessment and imaging study were performed before the treatment, at discharge, and at follow-up.

### Outcome evaluation

The recanalization of CVST patients were evaluated by MRV at discharge and followed up at 6th month. The criteria for recanalization was as follows:(1) Complete recanalization was defined as all of the occluded sinus were completely recanalized ; (2) Partial recanalization was defined as the complete recanalization of some sinus but partial recanalization of other sinuses; (3) No recanalization was defined as that all the occluded sinuses failed to be recanalized. Clinical outcomes including mortality, complications, and functional outcome at the time of admission, discharge, and 6th month were monitored according to Modified Rankins score (mRS) The clinical efficacy can be divided into three categories: good outcomes (mRS 0–2), partial improvement (mRS 3–4 points) and poor outcomes (mRS 5–6 points).

## Result

### Baseline clinical data

A total of 227 CVST cases received interventional therapy, and 56 cases were combined with ICH. Of the 56 cases , 41 were female and 15 were male with a mean age of 31 years (range 15–46). The most common clinical presentation of CVST with ICH was headache (48/56, 85.7%). Other symptoms include seizures (26/56, 46.6%), conscious impairement (10/56, 17.8%). Detailed clinical manifestations are shown in Table 1. 51 of the 56 cases (91.1%) had at least one identifiable risk factor. The main risk factors were pregnancy, puerperium and oral contraceptives. Detailed risk factors are shown in Table 2.

**Table 1:** Clinical manifestations of 56 patients.

Clinical manifestation	Numbers
Headache	48 (86.54%)
Hemiparalysis	12 (53.21%)
Epilepsy	26 (15.38%)
Disorder of consciousness	10 (10.90%)
Vision disorder	4 (9.62%)
Aphasia	3 (8.66%)

**Table 2:** Detailed risk factors of 56 patients.

Risk factor	Numbers
Pregnancy and puerperium	19 (33.9%)
oral contraceptives	12 (21.4%)
Infection	11 (19.6%)
Dehydration	5 (8.9%)
Endocrine diseases	2 (3.6%)
Nephrotic syndrome	2 (3.6%)
Unknown reason	5 (8.9%)

### Distribution of venous sinus thrombosis

The most common CVST sites were the superior sagittal sinus(SSS) and transverse sinus (TS). Deep venous sinus system was involved in 2 cases , and cortical veins were involved in 3 cases. 43 cases had involvement of more than one venous sinus. Distribution of venous sinus thrombosis has been summarized in Table 3.

**Table 3:** Distribution of venous sinus thrombosis of 56 patients.

Sinus involved	Number
SSS + TS + SigS	25
SSS + TS	10
SSS	8
TS + SigS	6
StrS	2
Cortical vein	3
StrS + TS	1
SSS + TS + SigS + StrS	1

SSS, superior sagittal sinus; TS, transverse sinus; SigS, sigmoid sinus; StrS, straight sinus.

### Imaging findings of cerebral hemorrhage

**Figure 1:**
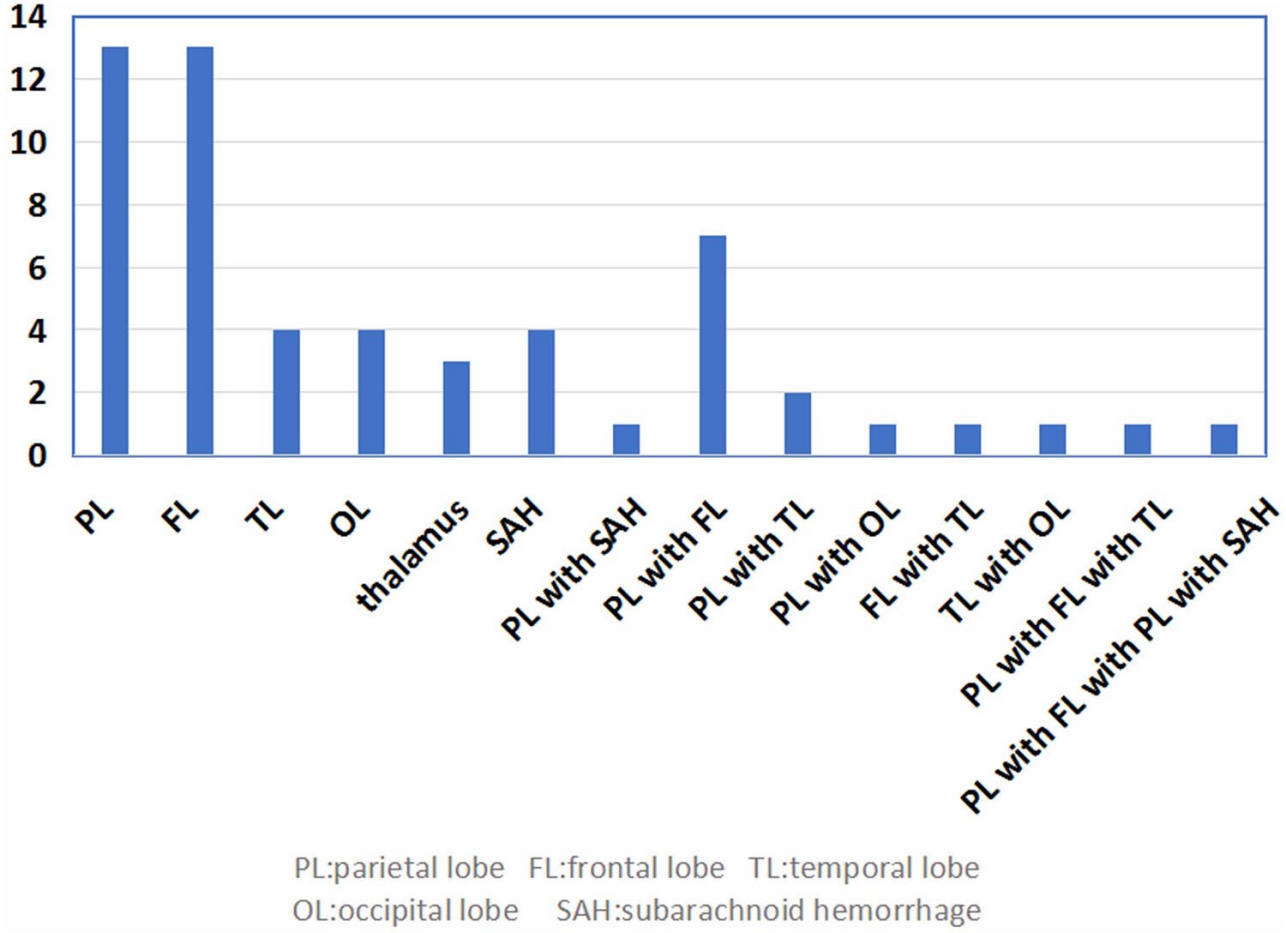
Localization and number of hemorrhage in 56 cases with CVST.

**Figure 2:**
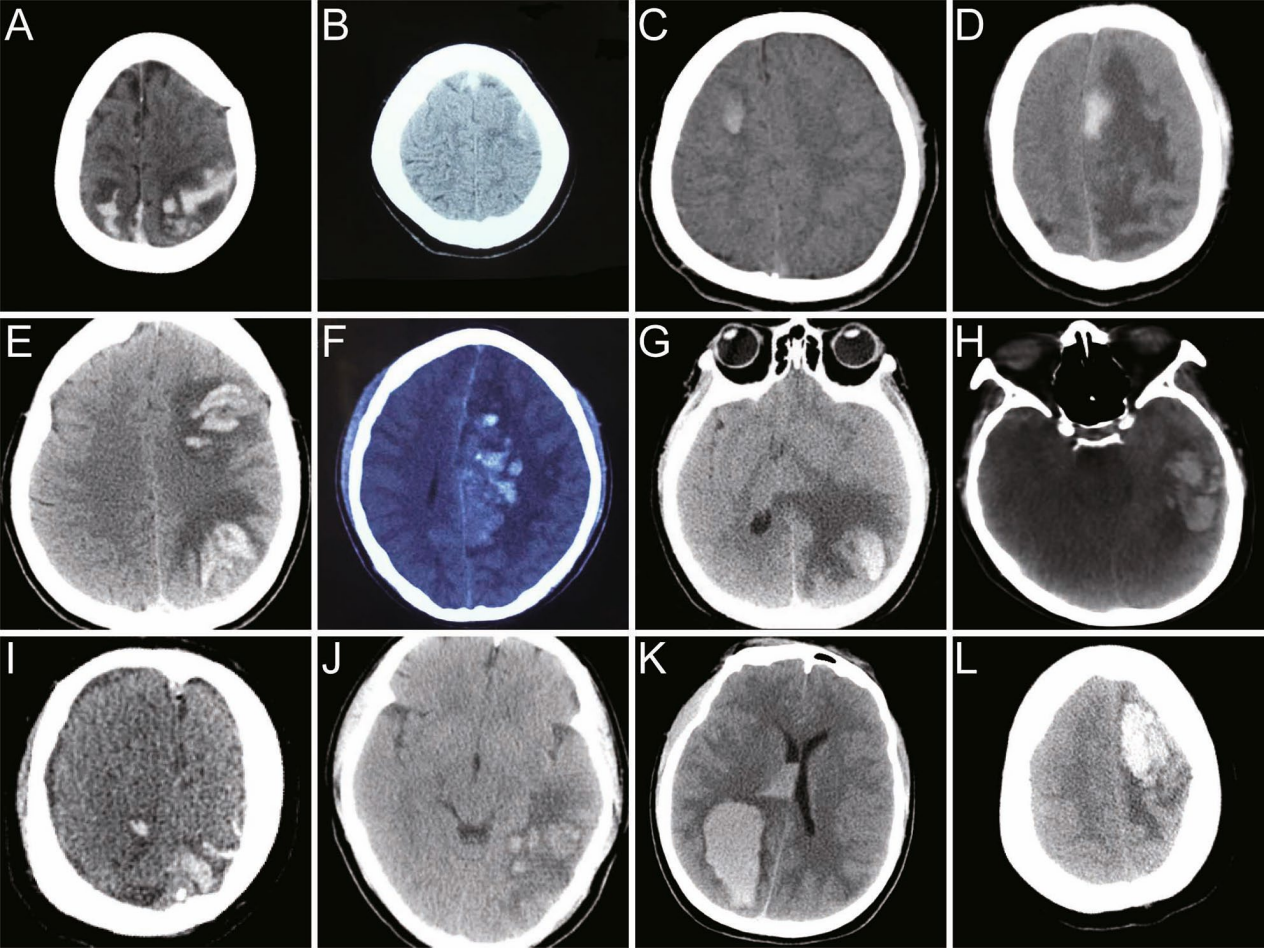
Serial common hemorrhagic neuroimaging findings of CVST cases.

Totally 56 cases had evidence of cerebral hemorrhage. CT scanning was the first imaging study in 52 cases (96%) and magnetic resonance imaging (MRI) in the remaining 4 cases. The most common type of bleeding was intraparenchymal hemorrhage. The most common hemorrhage involved the parietal lobe (28/56, 50%) and frontal lobe(23/56, 41.1%). Multifocal cerebral bleeding was observed in many patients. The locations of the cerebral hemorrhages are presented in Fig. 1. The radiological findings of common hemorrhages are presented in Fig. 2.

### Endovascular therapy and Sinus recanalization

**Figure 3:**
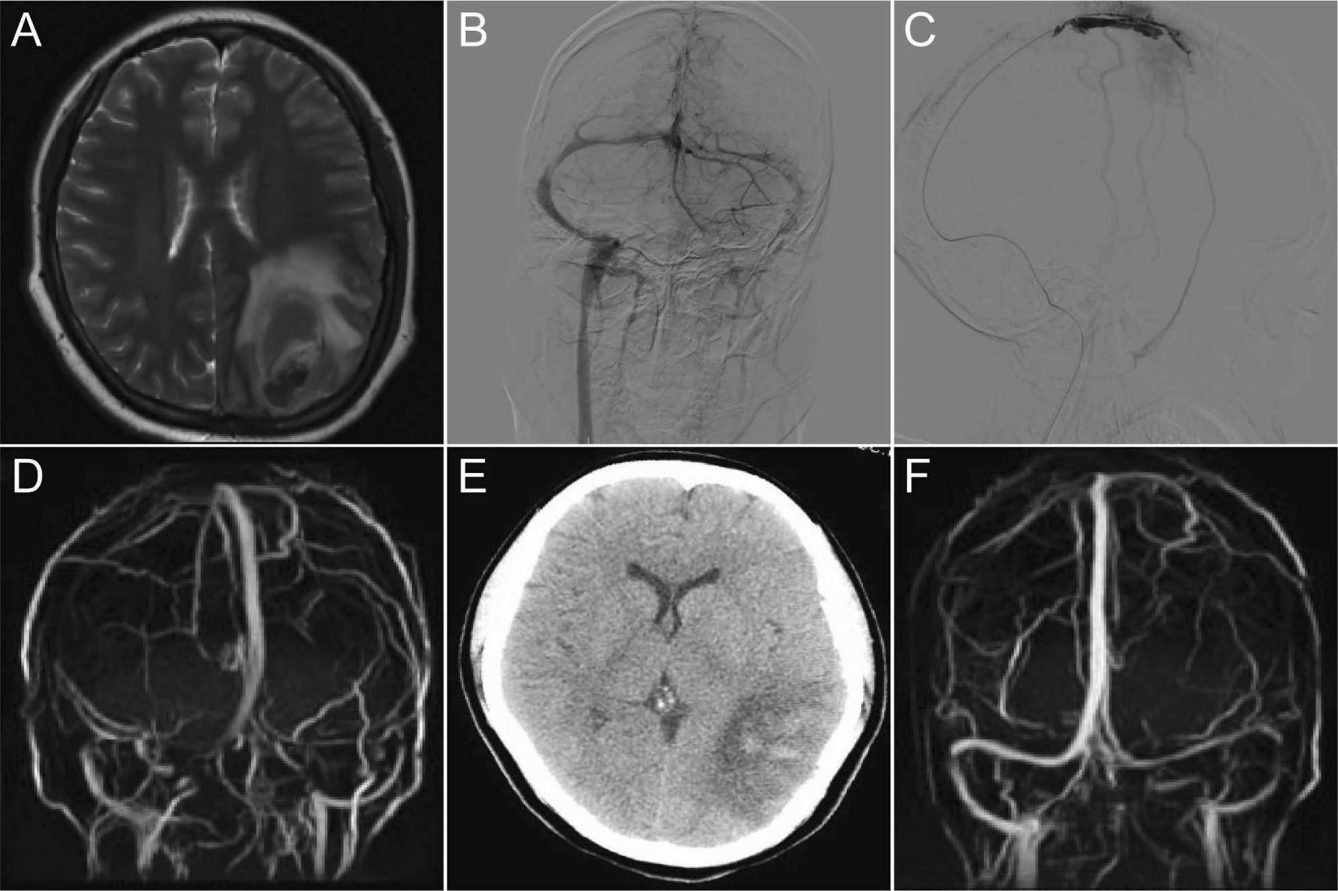
A representative 22-year-old female with CVST. MRI and DSA showed thrombosis of the superior sagittal sinus and left transverse sinus, accompanied by hemorrhage of the left occipital lobe (**A**,**B**). The intrasinus thrombolysis were performed (**C**). One week later, MRV showed recanalization of the superior sagittal sinus and the left transverse sinus (**D**), and CT scan showed absorption of hematoma (**E**). MRV showed complete recanalization of the superior sagittal sinus and the left transverse sinus at 3 months follow-up (**F**).

41 cases were treated only using IST, and the duration of thrombolysis was about 5–7 days (Fig. 3). One week later, MRV examination showed complete recanalization in 27 cases and partial recanalization in 12 cases. ICH exacerbation occurred in 4 cases during thrombolysis, including 3 cases with re-bleeding in new areas and one case re-bleeding at the primary site. For the 4 cases, the thrombolytic therapy was suspended and anticoagulation therapy was maintained. Decompressive craniectomy (DC) was performed in one of the 4 cases and eventually died. Two of the four cases eventually died while the other 2 cases survived with overall satisfactory outcomes. At discharge, 37/41(90.2%) cases had a mRS score (good) 0–2, 2 cases had a mRS score 3–4 while 2 (4.9%) died.

**Figure 4:**
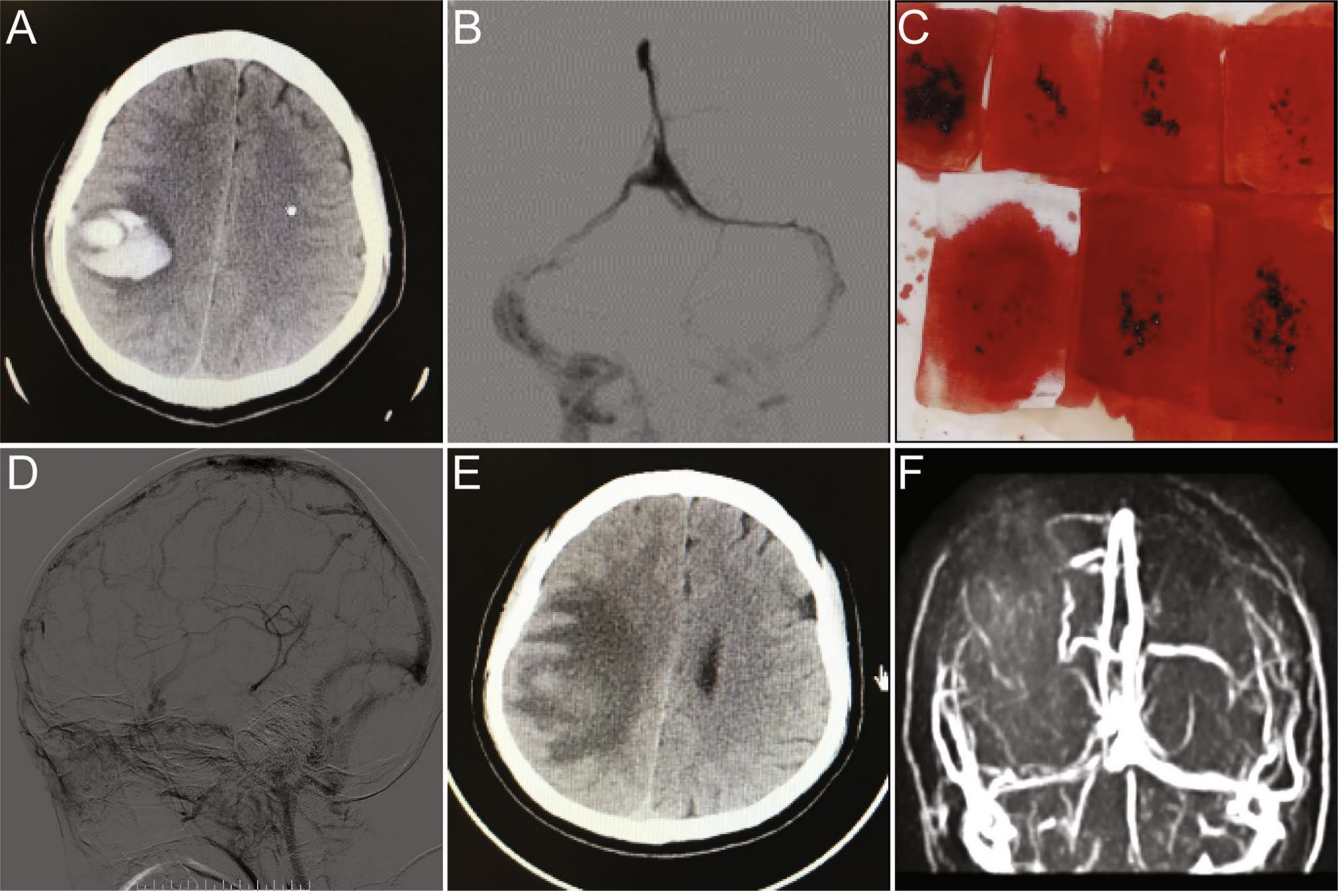
A representative 28-year-old female with CVST. CT showed left parietal lobe hematoma (A), and DSA showed superior sagittal sinus and bilateral transverse sinus thrombosis (**B**). Mechanical thrombectomy was performed using stent Retriever combined with local thrombolysis for one week,and a large number of red brown thrombi were removed during the operation (**C**). DSA reexamination showed partial recanalization of the superior sagittal sinus and sigmoid sinus (**D**). One week later, CT showed hematoma absorption (**E**).Three months later, MRV showed that the superior sagittal sinus and bilateral sigmoid sinus were unobstructed (**F**).

MT were performed in 15 cases (Fig. 4). Stent Retriever was used in 5 cases and a combined approach with aspiration catheter and stent retriever was conducted in 4 cases. Balloon thrombectomy was used in 3 cases, and aspiration catheter was used in 3 cases. All cases treated with mechanical thrombectomy were combined with local thrombolysis, and the duration of thrombolysis was about 5-7d. Worsening intracranial hemorrhage occurred in one patient, and decompressive craniectomy was performed following anticoagulant therapy. MRV examination showed complete recanalization in 11 cases and partial recanalization in 3 cases. One case died during thrombolysis due to acute cerebral hernia caused by brain swelling. At discharge, 12/15(80%) cases had a mRS score 0–2 and 1/15 (7.3%) died during thrombolysis. The other 2 cases were discharged with mRS score 3–4.

### Neurological status at discharge and long term outcome

At discharge, 49(87.5%) cases had a mRS score 0–2 and 3 (5.35%) died . The other 4 cases were discharged with mRS score 3–4. 51 cases were followed up at 6th month and two cases were not followed up at 6th month. MRV showed that 41 cases (80.39%) were completely recanalized, and 10 cases (19.61%) were partially recanalized. Among the 51 cases who were followed up, 49 cases had a mRS score of 0–2, and 2 cases had a mRS score of 3–4.

## Discussion

CVST is a rare type of stroke, and the mortality of CVST was about 10%-30%. The causes of neurological symptoms in CVST include: (1) progressive deterioration of intracranial hypertension as the result of occlusion of the venous sinuses and (2) progressively increased cerebral edema, venous infarction, and ICH. CVST associated with ICH becomes a special issue beacuse of different understanding of the treatment of CVST with ICH. The major therapeutic debates include IST or MT because of previous ICH and delayed hematoma enlargement related to pre-existing ICH, or treatment or both. ICH is the most important prognostic factor of poor outcome in CVST^7,13^. However, this subtype of CVST with ICH has never been systematically studied.

IST could make the occluded venous sinus recanalized rapidly, and improve the outcomes of CVST patients^8,14–16^. Among the cases treated with thrombolysis therapy alone in our study, two patients died because of worsening hematoma possibly related to thrombolysis. Complete recanalization was achieved in 27 of the 41 patients, and partial recanalization in 12 cases. Our study showed that IST improves clinical and radiological outcome for the CVST cases coupling with ICH, and ICH should not be regarded as a contraindication for thrombolysis.

MT can rapidly restore the venous sinus blood flow, and prevent the more significant conditions such as herniation and ICH^7,17^. However, previous studies have demonstrated that cerebral venous infarction might develop into brain hemorrhage^5,18^. Local thrombolysis may clear the remaining thrombus in venous sinus and recanalize the residual thrombosis in cortical veins, reducing the risk of venous hemorrhage. Our result suggest that MT combined with IST is a relatively effective and safe method to treat CVST with ICH.

**Figure 5:**
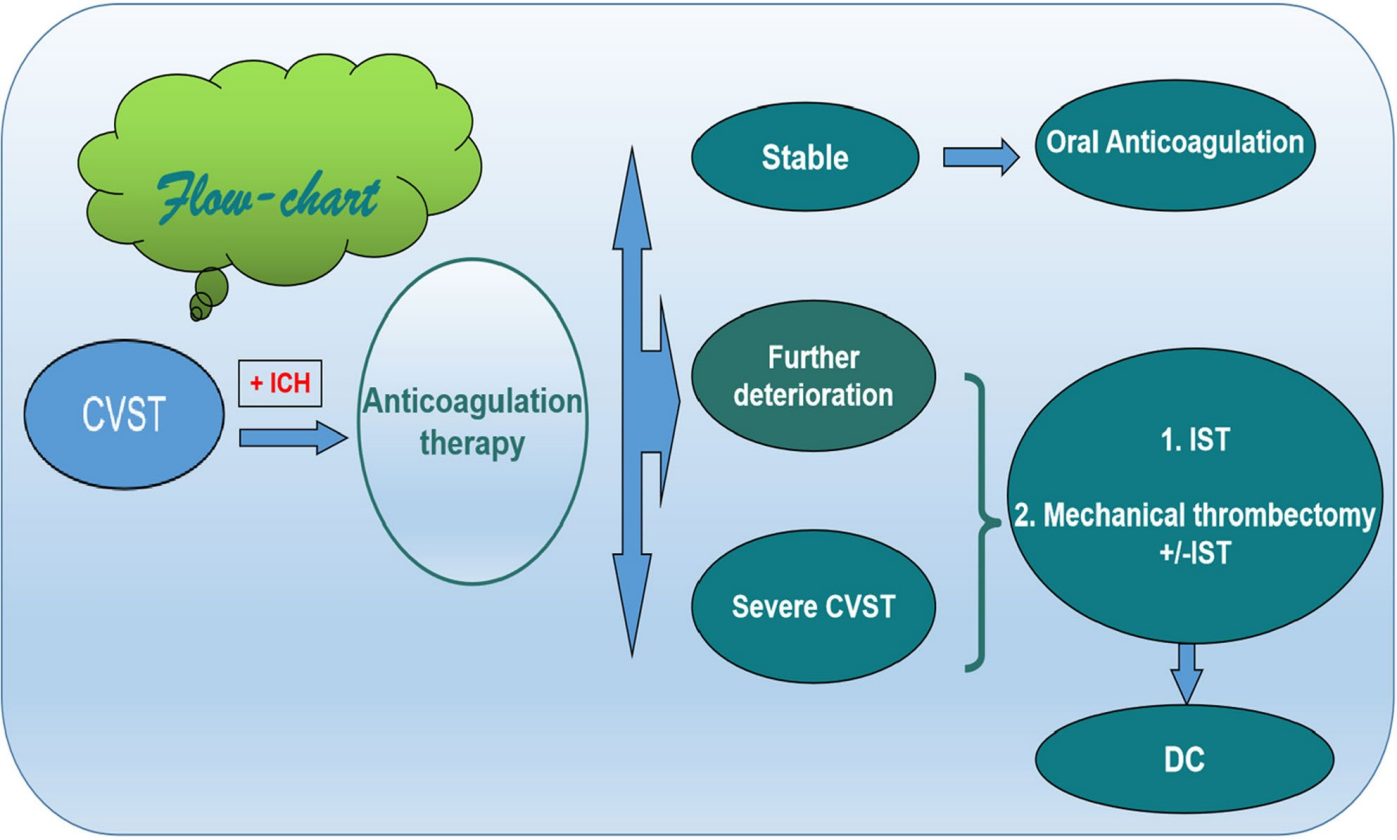
The treatment strategy flow-chart of CVST with ICH.

The results of the TO-ACT (Coutinho et al. JAMA Neurology 2020) showed that EVT did not appear to improve functional outcome of patients with CVT compared with standard medical care only^18^. But 185 patients with severe CVT included 42 studies coupling with pretreatment ICH in 60% and stupor or coma in 47% were treated with mechanical thrombectomy ,and overall, a good outcome was reported for 84%of patients^19^. We think future studies will demonstrate recovery rates after EVT. There are no specific recommendations about the timing and indication of anticoagulation, local thrombolysis and MT with or without IST for CVST. The timing and indication of endovascular therapy should be determined based on individual situation. The literature showed no significant differences between MT and IST^20^. In addition, the proceduralists have different understanding of the treatment of CVST with ICH because specific guidelines are not available currently. Based on our treatment experience, we proposed a flowchart for the treatment strategy of these patients (Fig. 5).

This study highlights the benefits of endovascular interventional therapy in patients of CVST with ICH. This case series has also demonstrated safety and effectiveness of endovascular interventional therapy. The main limitation of our study is that it is a retrospective study and there is no control group for comparison. We could not rule out the presence of treatment bias because the proceduralists prefer different treatment strategies without specific guidelines. Particularly, the timing to start endovascular therapy is complicated and should be assessed individually. Moreover, due to the relatively small samples size in the our study, further studies with large samples are warranted to further address the issues.

In conclusion, CVST with ICH is a special and controversial subgroup. The major controversy is whether IST or MT therapy should be carried out because of previous ICH and delayed hematoma exacerbation. Our data suggest that endovascular treatment may improve clinical and radiological outcome in most patients of CVST with ICH, but confirmation in prospective, controlled studies is warranted.

## Abbreviations

CVST: Cerebral venous sinus thrombosis
ICH: Intracranial hemorrhage
IST: Intrasinus thrombolysis
MT: Mechanical thrombectomy
DC: Decompressive craniectomy
MR: Magnetic resonance
DSA: Digital subtraction angiography
SSS: Superior sagittal sinus
TS: Transverse sinus
SigS: Sigmoid sinus
StrS: Straight sinus
